# Validating and reliability testing the descriptive data and three different disease diagnoses of the internet-based DOGRISK questionnaire

**DOI:** 10.1186/s12917-016-0658-z

**Published:** 2016-02-20

**Authors:** Johanna Roine, Liisa Uusitalo, Anna Hielm-Björkman

**Affiliations:** Faculty of Agriculture and Forestry, Department of Agricultural Sciences, University of Helsinki, 00014 University of Helsinki, P.O. Box 27, Helsinki, Finland; Faculty of Veterinary Medicine, Department of Equine and Small Animal Medicine, University of Helsinki, 00014 University of Helsinki, P.O. Box 57, Helsinki, Finland

**Keywords:** Dog, Internet-based questionnaire, Validation testing

## Abstract

**Background:**

The DOGRISK questionnaire is an internet-based ongoing study of canine nutrition, living environment, and disease. Here we aim to assess the performance of the questionnaire using data from the first three years in relation to some descriptive and disease variables. We used associated questions, official register records, test-retest repeatability, and email/mail contact with questionnaire respondents.

**Results:**

Reliability against an official register of gender, season of birth, breed, and results of hip radiography was tested and Cohen’s Kappa was between 0.95–0.99. Internal consistencies of hypothyroidism status and dog’s age were calculated using Cronbach’s Alpha (α = 0.95 and α = 0.99, respectively). Test-retest repeatability of ten variables among 224 participants was analyzed. Gender, season of birth, and born in owner family had Cohen’s Kappa > 0.86, color of coat, vaccination status as an adult, other dogs in household, and vaccination status as a puppy had Cohen’s Kappa between 0.67–0.80, and body condition score under two months of age and tidiness of household, had Cohen’s kappa of 0.45 and 0.42, respectively. In addition, time spent outside had Cohen’s kappa of 0.37. Of the owners contacted by email/mail to confirm their dog’s atopy/allergy (skin symptoms), 8.9 % reported that they had given an incorrect answer (positive predicted value 91 %), but only 69 % of all reaffirmed positive answers had a diagnosis set by a veterinarian.

**Conclusions:**

Our study showed that owners were diligent with basic information and with the status of three diseases. Cohen’s Kappa in the reliability of the test-retest was in most variables at least 0.67. We propose that the descriptive variables and the disease variables be used as such when we generate hypotheses from the DOGRISK data.

## Background

Internet-based data collection is a cost-effective and simple method to obtain epidemiological data, widely used in human research [1, 2]. With dogs, the use has been sporadic; the internet has been utilized in, for instance, cross-sectional studies of separation-related disorder [3], risk factors for surgical gastric dilatation-volvulus [4], and risk factors for injury among agility dogs [5]. A validated, large-scale, internet-based, longitudinal questionnaire for Labrador retriever health now exists in the UK [6, 7]. The DOGRISK questionnaire presented in this article differs from these earlier studies in that it contains multiple disease endpoints and is open to all dogs irrespective of their breed.

The Finnish DOGRISK questionnaire is an ongoing, large, internet-based, cross-sectional study of canine nutrition, living environment, and health that contains over 1300 variables. It was launched in December 2009 by the DOGRISK research group of the Department of Equine and Small Animal Medicine, University of Helsinki, Finland. The DOGRISK questionnaire aims to find associations between nutritional, descriptive, and environmental factors and diseases. It is based on the two-page paper-version pilot questionnaire from 2003 that attracted over 1000 answers. To ensure that all important food and diet, environment, and phenotype-related options would be included in the questionnaire, the face and content validity of the questionnaire was checked with veterinary colleagues and people from the dog food and supplement industry, dog breeders, active dog owners, and different dog interest groups. Draft versions were tested on volunteers in the waiting room of the Animal Hospital of the University of Helsinki. A nearly final 14-page version was tested in two clinical trials and during a week in June 2009 among veterinarians, animal nurses, and dog owners in our veterinary hospital waiting room. Before launching the final DOGRISK questionnaire (www.ruokintakysely.fi) in December 2009, also the internet version was tested on 27 dog owners. This questionnaire is the first to gather a vast information of diseases and diet of dogs meticulously over lifetime. The collected environmental data will be used in later nutritional analyses as adjusting background variables.

To collect useful, high-quality data, it is essential to have a carefully designed and well-validated questionnaire serving the specific purpose. Telephone questionnaires for measuring dogs’ dietary and living patterns, food intake, exercise, and health status [8, 9] have been validated previously. To enable hypotheses to be generated based on the associations derived from the data, the questionnaire must first be validated and tested for reliability. A questionnaire is a valid instrument if what it measures is what it was originally designed to measure. Validity can be evaluated as the quality and the overall comprehensiveness of the questionnaire, requiring time and effort during the development phases of the instrument. By contrast, reliable self-report instruments are defined by their consistency. There are mainly three things affecting the reliability of this type of questionnaire data: owner data entry errors, how the owner understands the questions, and the owner’s ability to remember incidents of interest [6]. Greater reliability will ensue when instructions for the completion of the questionnaire are clear and there are limited distractions in the testing environment [10].

The aim of this work was to describe the validity of owner-entered descriptive information and disease status comparing it with the same data from the official Finnish Kennel Club (FKC) register. In addition, the reliability was tested through internal consistency by comparing the answers to two related questions within the questionnaire, and by comparing owner-entered information about their dog having skin symptoms with responses to a short email questionnaire sent to owners. The test-retest repeatability was ascertained by using the questionnaires that were filled in twice for the same dog.

## Methods

Eligible respondents were all dog owners who were able to respond to the questionnaire in Finnish. They were recruited by letting dog owners know about the questionnaire at dog fairs, through dog clubs, in dog magazines, by interviews in the media, and later by sending out flyers with a raw food selling vending car. Up to March 23, 2013, a total of 8813 questionnaires had been filled in by Finnish dog owners. The study population consists of 261 different breeds and 1155 mixed-breed dogs (13.1 %) of all age groups, from puppies to senior dogs. The earliest date of birth in the sample was April 8, 1983, as owners of deceased dogs were also allowed to fill in the questionnaire. The study population consists of dogs from all over Finland (northern Europe). Not all questions were mandatory, so the respondents had the opportunity to leave the questions unanswered.

### Criterion validity: Inter-rater reliability against an official register

From the whole DOGRISK questionnaire study population, 487 dogs with an owner-entered official Finnish Kennel Club (FKC) canine registration number were chosen as a convenience sample, starting from the first dog and moving forward until the 487th. Breed, gender, date of birth, and results of official hip radiographs (if available) were confirmed from the official FKC register and compared with the answers given to the corresponding four questions in the DOGRISK questionnaire. Subject characteristics are shown in Table 1. The date of birth taken from the register was recoded into four variables to match the season answers given in the questionnaire: winter (from December to February), spring (from March to May), summer (from June to August), and autumn (from September to November) (Table 2). The hip radiograph results are in both the register and the DOGRISK questionnaire given as one of 25 possible variants from A/A to E/E according to the internationally used FCI grading system. These official hip dysplasia screening results are recorded as follows: A = no signs of canine hip dysplasia (CHD), B = near normal hip joints/borderline, C = mild CHD, D = moderate CHD, and E = severe CHD [11]. The first of the letters corresponds to the dog’s left hind leg and the second letter to the right. However, as most of the owners probably do not remember which leg was the worst, both the owners’ answer and the result from official register were recoded so that A/B and B/A indicated the same, B/C and C/B indicated the same, etc. Cohen’s Kappa (κ) was used to evaluate the reliability of the answers for all descriptive variables.

**Table 1:** Characteristics of the entire DOGRISK population and the two subpopulations used in the present article

Characteristics	Entire DOGRISK questionnaire cohort (*n* = 8813)	Subcohort from the Finnish Kennel Club register (*n* = 487)	*P*	Subcohort with double answers on the DOGRISK questionnaire (*n* = 244)
Age, years (±SD)	4.1 (±3.2)	4.1 (±2.9)	0.087	First answers: 3.3 (±2.9); second answers: 4.4 (±3.1)
Gender, % male	47.1	43.0	0.044	48.4
Number of different breeds	262	126		101

**Table 2:** Comparison of season of birth

DOGRISK	Finnish Kennel Club register			
	Winter	Spring	Summer	Autumn
Winter	101	6	0	3
Spring	1	163	1	0
Summer	0	0	116	1
Autumn	0	0	2	89

Answers of the owners about the season of birth of their dog from the DOGRISK questionnaire compared to the birthday data from the same dogs in the Finnish Kennel Club register (*n* = 483)

Winter, from December to February; Spring, from March to May; Summer, from June to August; Autumn, from September to November

### Internal consistency within the questionnaire

To assess the internal consistency in the owners’ answers, we compared two answers on the questionnaire that would always be expected to coincide. For this, we chose hypothyroidism, which is a common disease requiring specific diagnostic blood work that is done by veterinarians and that always needs medication. The questionnaire includes a question on whether the dog has the disease or not, and another question on ongoing medication (open question where owners were to fill in all medication used). Cronbach’s Alpha (α) was used to evaluate the reliability of the answers to the owner-reported disease status and medication.

The questionnaire also included a question on the dog’s age (with three response options: puppy/0-6 months, young/7-18 months, or adult/choose the age in years between 1 and 21 years) and another question on the date of birth. We calculated the difference between the date when the owner filled in the questionnaire and the birth date of the dog, and then recoded it into years: 0.1–0.5 as 0.25 year (puppy), 0.6–1.9 as 1 year (young dog), 2.0–2.9 as 2 years (adult), etc. We then compared this value to the owner’s answer of the dog’s age in years using Cronbach’s Alpha.

### Reliability against additional questions

All dog owners who had answered ‘yes’ to the question ‘Does your dog suffer from atopy/allergy (skin symptoms)?’ and had provided either their email address or street address were contacted by email/mail (*n* = 1354) and asked whether their original answer was correct and whether the condition had been diagnosed by a veterinarian. The owners also had the opportunity to explain their dog’s symptoms more thoroughly.

Additionally, all dog owners who had not ticked an answer ‘yes/no’ to the question on whether their dogs suffered from atopy/allergy (skin symptoms) (AASS) but had answered one or more of the related questions (has had it rarely/has had it often; started at the age of; is still having the disease; the disease cured after changing the diet/ I haven’t noticed that the diet change helped) were also contacted by email/mail (*n* = 197) and asked whether their dog suffered from the disease or not. Positive predictive value (PPV) was calculated to evaluate the percentage of true positives of all positive answers of owner-reported disease status of AASS. Here the correct positive answer to the question ‘Does your dog suffer from atopy/allergies (skin symptoms)?’ reaffirmed by the owner by email/mail was used as the ‘golden standard’. From all owner-reaffirmed positive answers, the percentage of dogs with a diagnosis set by a veterinarian was calculated.

### Repeatability by a spontaneous, non-prospective test-retest

There were 244 owners in the study sample who had answered the questionnaire twice of their own initiative, three who had answered it 3 times, and one who had answered it 4 times; in the analyses, we used only the twice-answered questionnaires. For some questions, the owner might not have had all information at hand when they filled in the questionnaire the first time. In the questionnaire pre-text, the respondents had indeed been instructed not to submit the questionnaire before it was completely ready, but instead to save the answers temporarily. However, some owners might have submitted the questionnaire before they contacted the breeder and then resubmitted a new questionnaire after receiving the answers from the breeder. Because of adding new information to the questionnaire, we could not use this test-retest data to analyze the repeatability of questions on diseases or of many of the living conditions. Repeatability of ten questions was analyzed using Cohen’s kappa; these included gender, season of birth, color of coat, born in owner family, body condition score under two months of age, time spent outside under two months of age, vaccinations as a puppy, tidiness of household, vaccinations as an adult, and other dogs in household. When calculating the kappa for adult vaccinations, only dogs aged over 18 months were included.

## Results

The characteristics of the DOGRISK study population and the subpopulations from the FKC and the retest sample are shown in Table 1. The three most prevalent breeds in the entire DOGRISK study population were mixed breed dogs (13.1 %), German shepherd dog (6.1 %), and Labrador retriever (3.4 %). In the FKC subcohort they were German shepherd dog (7.6 %), Finnish Lapphund (4.3 %), and Shetland sheepdog (3.7 %), and in the subcohort of retest sample mixed breed dogs (8.2 %), German shepherd dog (8.2 %), and Rottweiler (5.3 %).

### Criterion validity: Inter-rater reliability against an official register

Reliability of season of birth, gender, breed, and results of hip radiography when DOGRISK questionnaire answers were compared with official FKC register data was following: κ = 0.96, κ = 0.99, κ = 0.99, and κ = 0.95, respectively. Results are shown in Table 3.

**Table 3:** Reliability of questions in the DOGRISK questionnaire

Question	Kappa statistic	95 % CI	n
Season of birth	0.96	0.94–0.98	483
Gender	0.99	0.98–1.00	468
Breed	0.99	0.98–1.00	478
Hip radiograph results	0.95	0.91–0.99	115

Comparing owners’ answers in the DOGRISK questionnaire with the same data in the Finnish Kennel Club register using Cohen’s Kappa

### Internal consistency of the questionnaire

The owner-reported hypothyroidism diagnosis and ongoing hypothyroid medication of the dog were compared and the Cronbach’s α was 0.95 (*n* = 8081). In the consistency between the dog’s age and date of birth the Cronbach’s α was 0.99 (*n* = 3540).

### Reliability against additional questions

Altogether 515 (38 %) of 1354 owners who had responded ‘yes, my dog suffers from atopy/allergy (skin symptoms)’ (AASS) also answered our email/mail. Of these, 457 (89 %) reported having given a correct answer and 58 (11 %) an incorrect answer. All 58 answers reported as incorrect were controlled one by one, and based on the owners’ explanation, 11 were kept as a ‘yes’ and the rest were considered false positives (*n* = 47). The PPV of the owner-reported disease status of AASS was 0.91. Thirty-seven of the 47 answers were excluded from future analyses as they could not be categorized as atopic nor healthy.

Of the 197 owners who had ticked only one of the AASS-specifying questions (when diagnosed, if symptoms occur frequently, etc.), but not the disease question itself, 63 (32 %) answered our email/mail; 49 (78 %) reported that their dog had AASS, while 14 (22 %) reported that their dog did not have AASS. The 49 were recoded as ‘yes’ into the questionnaire data. The 14 ‘no’ answers were controlled one by one, and based on the owners’ explanation, one was recoded into ‘yes’, three were recoded into ‘no’, and ten could not be assigned to either category. For the owners did not answer the email (*n* = 134), this question was left empty on the questionnaire as these dogs also did not have any answer to the main question of ‘Does your dog suffer from AASS?’.

After these checks, 1357 (1354–47 + 49 + 1) positive answers remained in the data, with a total of 518 (457 + 11 + 49 + 1) confirmed answers (38 %). In addition, 228 dogs had positive answers without any contact information for the owner. Of the confirmed positive answers, only 69 % had a diagnosis set by a veterinarian.

### Repeatability by a spontaneous test-retest

Altogether 244 owners had filled in the questionnaire twice. The time period between the answers varied from 1 day to 38 months. Three variables, i.e. gender, born in owner family, and season of birth, had Cohen’s kappa between 0.86–0.96. Four variables, color of coat, vaccination status as an adult, other dogs in household, and vaccination status as a puppy, had Cohen’s kappa between 0.67–0.80. Two variables, body condition score under two months of age and tidiness of household, had Cohen’s kappa of 0.45 and 0.42, respectively. Time spent outside under two months of age had Cohen’s kappa of 0.37. Results are presented in Table 4.

**Table 4:** Test-retest repeatability of ten questions in the DOGRISK questionnaire

Question	Kappa statistic	95 % CI	n
Constant			
Gender	0.96	0.92–0.99	241
Season of birth	0.86	0.81–0.91	234
Color of coat	0.80	0.73–0.87	218
Born in owner family	0.96	0.89–1.04	244
Possibly not known when first responding			
Body condition score when dog aged under 2 months	0.45	0.28–0.62	105
Time spent outside when dog aged under 2 months	0.37	0.26–0.49	118
Vaccinations as a puppy	0.67	0.048–1.28	230
Possibly changing with time			
Tidiness of household	0.42	0.29–0.54	241
Vaccinations as an adult (only dogs aged over 18 months included)	0.76	0.50–1.02	147
Other dogs in household	0.71	0.60–0.81	198

Comparing the repeatability of the DOGRISK questionnaire using Cohen’s Kappa in a subpopulation of dogs whose owners filled the questionnaire twice

Consent from the dog owners’

In the questionnaire the dog owners’ are informed that the results will be published in national and international journals. By filling in the questionnaire they give their consent to this

Link to the questionnaire

The questionnaire can be found from www.ruokintakysely.fi (only in Finnish)

## Discussion

Since we have 43 disease diagnoses (117 when including all drop-down lists) in the DOGRISK questionnaire, it was not feasible to validate them all. However, as we are interested in using different diseases in our future analyses, we chose to validate one medicated disease that could be evaluated easily using a concurrent medication question (hypothyroidism), one diagnosis that could be compared with an external official register (canine hip dysplasia; CHD), and one disease that even for a specialist is considered difficult to diagnose with 100 % certainty (canine atopic dermatitis or atopy), which in the questionnaire was covered by a wider question: atopy/allergy (skin symptoms)(AASS). The internal consistency for hypothyroidism was excellent (α = 0.95, *n* = 8081). This was expected since hypothyroidism is also a human disease and it can be speculated that people may remember it more easily because of this. It is also always diagnosed from a blood sample at a veterinary clinic, and it invariably requires medication. Discrepant answers to the two questions on diagnosis and medication were very few, but could arise from owners having just visited the clinic and awaiting results, which would have yielded a ‘yes’ for diagnosis but nothing for medication. Another possibility might be that the owner could not remember the name of the disease or the name of the medication, resulting in one of the questions left unanswered.

The reliability of the radiographic CHD results was also excellent (*κ* = 0.95). Many dog breed associations in Finland take part in a national hip screening program (PEVISA) [12] where dogs’ hips are screened by radiographs according to the Fédération Cynologique Internationale (FCI) [11] at the age of 12-18 months. The screening result is expressed as a letter, according to their worst hip; A (healthy), B, C, D or E (severe CHD). The small differences in owner-reported CHD results and the register data might reflect the fact that some owners take new radiographs when their animals are older to see if they have improved (or worsened). This would yield an inconsistency in the validation, as we have old results in the questionnaire but new results in the FCI register. Also, owners might simply forget the letters. We only looked at dogs that had official hip radiograph results, meaning that they were pure-bred dogs aged over 18 months.

Atopy/allergy (skin symptoms) (AASS) is much more difficult to diagnose than hypothyroidism and CHD, and therefore, we expected its reliability to be much lower than for the other two diagnoses. The diagnosing protocol is time-consuming and includes numerous treatment trials, elimination diet trials, blood work, etc. This might be too extensive and expensive for many owners. Thus, many pruritic dogs are incorrectly believed to be atopic by owners or even veterinarians, who sometimes also find it difficult to diagnose atopy and allergies. Some dogs therefore probably have the disease but lack a confirmed diagnosis. The dog’s symptoms also might have disappeared due to environmental or dietary changes, or they may be so mild that the owners think that their dog is healthy. For this validation, we sent a short email/mail questionnaire to the owners to ask about their dogs’ diagnosis. Altogether 31 % of the owners reported that the diagnosis was not verified by a veterinary work-up. However, response consistency or disease repeatability was good: the PPV was 0.91 when comparing the answers in the DOGRISK questionnaire and in the email/mail questionnaire. Therefore, we may consider the owner-reported disease status of AASS quite reliable, bearing in mind the difficulties in diagnosing this disease. Because email/mail was not sent to owners reporting that their dogs did not suffer from AASS, we could not conclude how many true- or false-negatives we had.

Owners seemed to be diligent with basic information, as the internal consistency of two questions related to the dog’s age was excellent. Also data on gender, season of birth, and breed matched very well with the official register records. Because all dogs in these analyses were pure bred dogs, the new owner should have received the registration book from the breeder or previous owner in which this information can be verified. In fact, most of our data concerning descriptive information and diseases can be considered good or excellent. Only data for the time before the owner had the dog can be considered slightly more unreliable.

Data on web-based questionnaire validity and reliability among human subjects are vast, and a comprehensive review is not feasible in this context. Although between-study comparisons are complicated by differences in the study methods and statistical measures used, the DOGRISK questionnaire appears to compare well with results for humans. The reliability is highly dependent on the exact variable analyzed, as shown by Stanton et al. [13], who found Kappa coefficients ranging from 0.65 to 1.00 for sociodemographic variables and from 0.21 to 0.71 for health variables. McAlindon et al. [14] compared consistency between answers given to age and date of birth and found a discrepancy rate of 1.3 %, similar to our figure of 1.0 % (*n* = 3540). Upon comparing self-reported hip osteoarthritis with results of clinical examination, Ratzlaff et al. [15] reported a positive predictive value of 61 %, a negative predictive value of 98 %, a sensitivity of 81 %, and a specificity of 94 %.

The retest population was not a planned repeatability study population, but a subgroup of the group responding to the existing questionnaire twice of their own initiative. Utilizing the responses of the owners who had answered the questionnaire twice was a limitation in our study, as all data will not necessarily be the same. Answers for gender, color of coat, season of birth, and born in owner family are considered stable since they should not change with repeated response, and repeatability in these was indeed high, being very similar to that in Sallander et al. [8].

We provided an option in the questionnaire to save answers midway through the answering process without submitting the questionnaire and to continue answering later. For those that used this option, it probably had a positive effect on the reliability of the answers, as respondents could gather all information before submitting the questionnaire and they could fill in the questionnaire at an optimal time as opposed to completing it in a rush. However, we also had informed respondents who contacted us by email that they could resubmit a totally new questionnaire at will if they received new information about the puppy stage or if conditions changed. As the two answers will then not be similar by default, they will show weaker repeatability. The most logical reasons for why some owners answered the questionnaire twice are because they had reported something incorrectly the first time, because they had forgotten that they had already filled in the questionnaire, because something had changed in their dog’s lives, because they wanted to report extra information about something they had no knowledge of the first time, or because other person in the household had responded to the questionnaire at the second time-point. The questions on body condition score and time spent outside, both covering the period when dogs were under the age of two months, could have been difficult for the owner to report since during this period puppies typically reside with the breeder. Thus, owners likely first provided an “educated guess” on the topic, followed by the actual answer given to them by the breeder, or a second own “educated guess”. This would explain the low repeatability in the answers to these questions, with answers to the question on puppy vaccination being somewhat more reliable, as it is common in Finland to vaccinate all puppies, and this information is usually given in written form (in the passport or vaccination booklet) to new owners if they adopt an older dog.

Questions about the environment were also deemed ill-suited to repeated questioning since owners might have moved, taken new pets, stopped smoking, etc., but we nevertheless chose to compare the following non-stable questions: tidiness of household, adult dog’s vaccinations, and other dogs in household. The time factor will be the reason for any discrepancy in the answers here, e.g. tidiness of the household may vary according to the life situation. Also, the number of dogs in the household may vary with time, as old dogs die and new puppies arrive. For the future analyses these variables can still be used but should be interpreted carefully.

## Conclusions

An epidemiological study, such as the DOGRISK questionnaire, with a large study population can be useful for searching for associations between diseases, nutrition, and descriptive and environmental factors, but the validity and repeatability of the questionnaire must be tested before any conclusions can be drawn. This study validated and tested disease diagnoses and descriptive and environmental factors for reliability; comparing data against that of an official register showed excellent agreement and internal consistency of the questionnaire was very good. The test-retest repeatability was substantial in few questions but good in most of the questions. We propose that the descriptive variables and the disease variables be used as such to generate hypotheses from the DOGRISK data and that second answers be used if owners have answered twice. The food and diet-related questions will be validated in a separate article.
